# Supplementary material for: ERG and Behavioral CFF in Light-Damaged Albino Rats
Source: Int J Mol Sci. 2022 Apr 8;23(8):4127. doi: 10.3390/ijms23084127 (PMC9027716; doi:10.3390/ijms23084127)
Supplement: Supplementary file 1 [file ijms-23-04127-s001.zip › ijms-1576224-supplementary.pdf]

International Journal of *Molecular Sciences*

MDPI

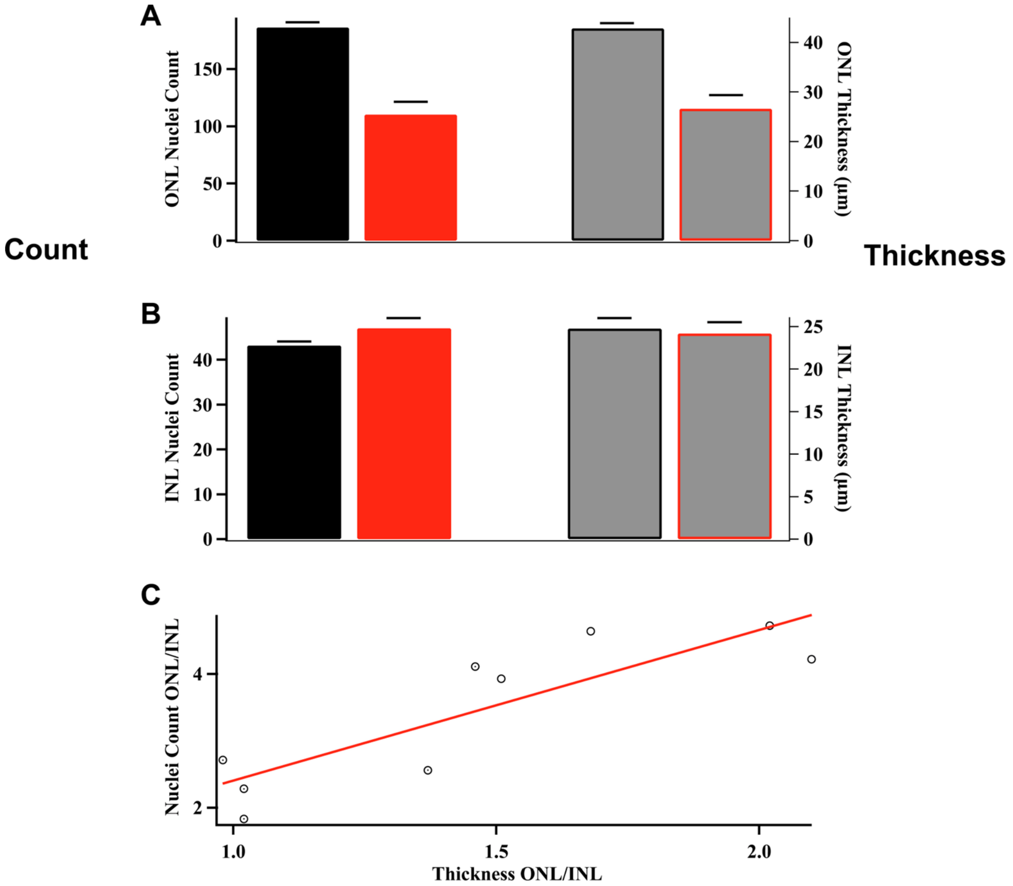

Figure S1. Correlation between nuclei counts and thickness measurements. The nuclei count and thickness measurements of ONL (A) and INL (B) at approximately 1.3mm inferior to ONH center were compared. Nuclei counts and thickness measurements were calculated from histological slices (5µm thickness) at R30 (n=4) and C30 (n=5). Black, control. Red, light damage. Error bar, SE. (C) The nuclei count ONL/INL ratio is significantly correlated with the thickness measurement ONL/INL ratio (Pearson Correlation coefficient = 0.86). Correlation is significant at the 0.01 level (P=0.003, 2-tailed, n=9). Thus, thickness measurements from the ONL and INL accurately represent nuclei counts.

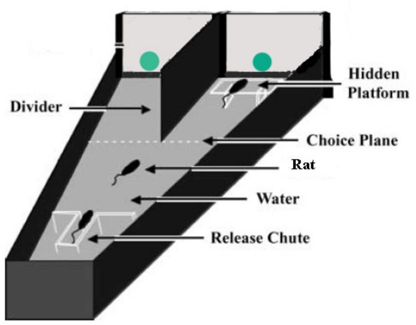

Figure S2. Behavioral testing apparatus. Overall length 140 cm. An opaque dividing wall defined the position at which choice of sides was determined.
